# Radiomorphologic profiles of nonsyndromic sagittal craniosynostosis

**DOI:** 10.1007/s00381-023-05998-x

**Published:** 2023-05-27

**Authors:** Tymon Skadorwa, Olga Wierzbieniec, Kamila Sośnicka, Klaudia Podkowa

**Affiliations:** 1 Department of Pediatric Neurosurgery, Bogdanowicz Memorial Hospital for Children, 4/24 Nieklanska St, 03924 Warsaw, Poland; 2 Department of Descriptive and Clinical Anatomy, The Medical University of Warsaw, 5 Chalubinskiego St, 02004 Warsaw, Poland

**Keywords:** Craniosynostoses, Children, Scaphocephaly, Infants

## Abstract

**Purpose:**

Numerous classification systems of nonsyndromic sagittal craniosynostosis (NSC) are applied but none has gained a wide acceptance, since each classification is focused on distinct aspects of cranial dysmorphology. The goal of this study was to depict the most common combinations of radiomorphologic characteristics of NSC and to separate groups where the patients were morphologically similar to one another and at the same time significantly different from others.

**Methods:**

The study was conducted on anonymized thin-cut CT scans of 131 children with NSC aged 1–12 months (mean age 5.42 months). The type of cranial dysmorphology was assessed using four criteria: skull shape, pattern of sagittal suture fusion, morphologic features and cerebrospinal fluid (CSF) spaces alterations. After assigning the categories, an unsupervised k-modes clustering algorithm was applied to identify distinct patients clusters representing radiomorphologic profiles determined by investigated characteristics.

**Results:**

Cluster analysis revealed three distinct radiomorphologic profiles including the most common combinations of features. The profiles were not influenced by sex nor age but were significantly determined by skull shape (V = 0.58, *P* < 0.0001), morphologic features (V = 0.50, *P* < 0.0001) and pattern of sagittal suture fusion (V = 0.47, *P* < 0.0001). CSF alterations did not significantly correlate with the profiles (*P* = 0.3585).

**Conclusion:**

NSC is a mosaic of radiologic and morphologic features. The internal diversity of NSC results in dissimilar groups of patients defined by unique combinations of radiomorphologic characteristics, from which the skull shape is the most differentiating factor. Radiomorphologic profiles support the idea of clinical trials targeted at more selective outcomes assessment.

**Supplementary Information:**

The online version contains supplementary material available at 10.1007/s00381-023-05998-x.

## Introduction

Despite several clinical and scientific approaches, nonsyndromic sagittal craniosynostosis (NSC) still remains a heterogeneous entity, investigated from various perspectives. Current research highlights not only its morphology, but also genetic, neurologic and developmental aspects [1–3]. Attempts are made to identify NSC-specific biomarkers, i.e. factors characteristic for the pathophysiology of this disease, able to determine its clinical subtypes or treatment effects [4]. These could be reproducible morphologic, functional or developmental criteria, such as cosmetic effect, cognitive disorders or speech development [5, 6]. However, an internal diversity of NSC and numerous endpoints contributed to the production of scattered data, selectively discussing morphologic, surgical or neurodevelopmental outcomes [7–11].

The heterogeneity of NSC has been demonstrated in many studies [12, 13]. In addition, new articles, analyzing the morphology of selected subtypes are appearing in the literature [14]. Regarding this tendency, it seems that the identification of NSC-specific characteristics would provide more homogenous groups of patients to facilitate robust clinical trials, what has been encouraged by the call for an international collaboration, announced in May 2022 [15].

However, the identification of homogenous NSC subtypes still remains complicated, since there exist numerous classifications of NSC, based on various morphologic and radiologic criteria, such as: skull shape [12], pattern of sagittal suture fusion [16], the presence of leading morphologic features [17] or the alterations of cerebrospinal fluid (CSF) spaces [18]. In addition, morphologic criteria of NSC often overlap with their radiologic determinants. Regarding these facts, the aims of this study were (1) to identify typical radiomorphologic characteristics of NSC and to separate groups where the patients were similar to one another and at the same time significantly different from others, (2) to determine how the NSC subtypes differ one from another, and (3) to determine which of the studied characteristics has the greatest impact on separating the subtypes and may be the basis for subsequent clinical analyses.

## Patients and methods

### Study design

This retrospective study was performed on anonymized digital data. No human subject was directly involved and consent to participate was not required by the protocol. All patients consented to collect the medical data in writing. This retrospective study was approved by the institutional Bioethics Committee (decision number AKBE/110/2021), and abides by the 1964 Helsinki Declaration and its later amendments or comparable ethical standards.

### Population

The group of patients with NSC aged 1–12 months included 131 consecutive children (97 boys, 34 girls) treated in our institution between 2010 and 2020. All patients had preoperative CT scans (low dose protocol). Mean age of the studied group was 5.42 months, standard deviation 2.51. Sex ratio was 2.85.

### Method

The study was performed using preoperative thin cut CT scans provided with Siemens Somatom Emotion with parameters: slice thickness 0.5 mm; the exposition was performed with source voltage 270 kV and current of 100 mA. All scans were analyzed with RadiAnt DICOM Viewer PL version 2021.2.2. (64-bit).

**Fig. 1:**
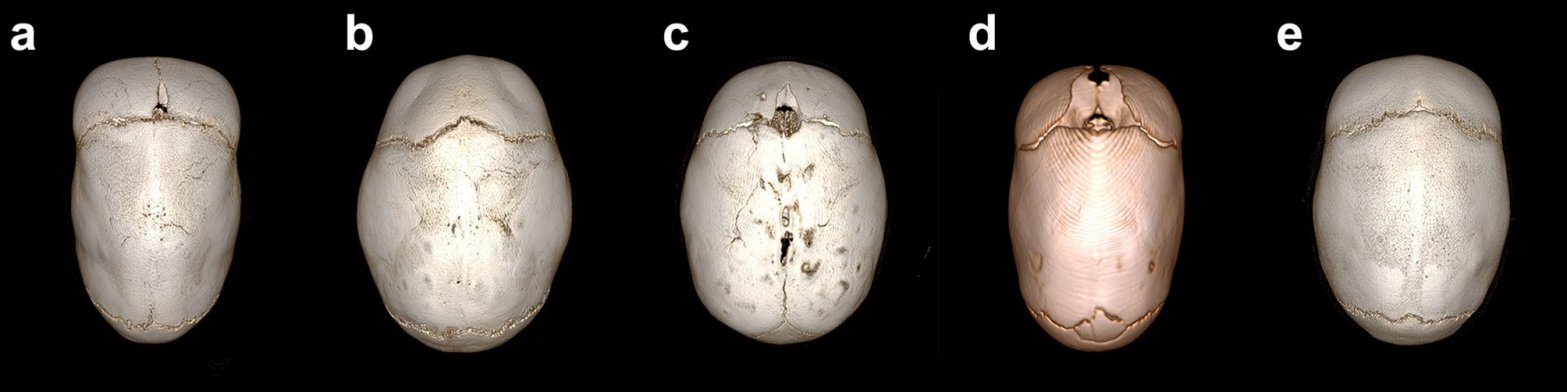
Shapes of the skull assessed in the study: **a** sphenocephaly; **b** clinocephaly; **c** bathrocephaly; **d** leptocephaly; **e** dolichocephaly

**Fig. 2:**
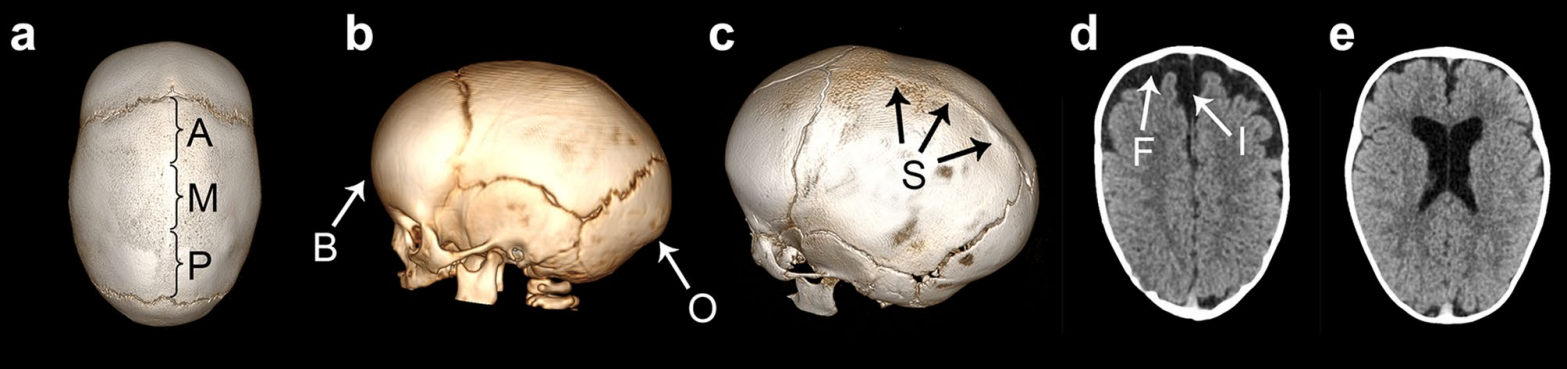
Radiomorphologic characteristics assessed in the study: **a** pattern of sagittal suture fusion (*A*, *M*, *P* indicate the portions of the suture); **b**, **c** morphologic features (*B* – frontal bossing; *O* – occipital bullet; *S* – sagittal ridge); **d**, **e** CSF spaces alterations (**d** – widening of frontal subarachnoid spaces (F) or interhemispheric fissure (I), **e** – ventriculomegaly)

Four radiomorphologic characteristics of NSC were assessed in each patient:

1. Shape of the skull (Fig. 1) – as provided by Di Rocco et al. [12], adapted from classical descriptions [19, 20]. Each patient was assigned to one category: sphenocephaly (with forehead width exceeding the interparietal diameter), clinocephaly (with retrocoronal depression), bathrocephaly (with occipital bulge), leptocephaly (equal narrowing of the skull) and dolichocephaly (undetermined or the combination of the features above). We introduced one modification in relation to Di Rocco et al. [12], that the types were not dependent on the pattern of sagittal suture fusion.
2. Pattern of sagittal suture fusion (Fig. 2a) – the fusion was assessed independently at each third portion of the sagittal suture (A – anterior, M – middle, P – posterior). The patterns were named after the fused portions, e.g. MP – fusion of middle and posterior thirds, AMP – fusion of the whole suture, etc.
3. Morphologic features (Fig. 2b–c) – we assessed the presence or absence of three features: S – sagittal ridge, O – occipital bullet, B – frontal bossing. Each patient was assigned a combination of the present features, e.g. SOB – when all three features were present, OB – when occipital bullet and frontal bossing were observed but not the sagittal ridge, etc.
4. CSF spaces alterations (Fig. 2d–e) – defined after Diab et al. [18] as a widening of the subarachnoid space and/or ventriculomegaly. The subarachnoid space was assessed in frontal regions (F) and in the anterior portion of longitudinal cerebral (interhemispheric) fissure (I)—in both regions the width > 5 mm was considered as widened. Ventriculomegaly (V) was calculated by Evans index > 0.3. Each patient was assigned a combination including observed CSF alterations, e.g. FV – frontal subarachnoid widening and ventriculomegaly, FIV – all the three alterations present, etc.

The above NSC characteristics were evaluated by two researchers independently with an inter-rater reliability of 84.6% (kappa 0.786, 95% CI 0.703–0.870, *P* < 0.01). When the choices of the two observers did not correspond, the researchers discussed them before the final distribution in order to reach consensus and avoid an observational bias.

In the next step, an unsupervised k-modes clustering was applied to identify distinct radiomorphologic profiles of NSC. We used k-modes clustering, as proposed by Huang [21], instead of k-means or hierarchical clustering as obtained data were categorical. K-modes clustering used an unweighted algorithm with a maximum of 10 iterations. After clustering, a multivariate analysis was performed to explore the relations among clusters. The differences between the variables were assessed with the chi-squared test. Effect sizes were measured with Pearson’s r or Cramer’s V. Probability values below 0.05 were considered statistically significant. The statistical analysis was performed with TIBCO Data Science/Statistica software by StatSoft Europe, version 13.3 PL for Microsoft Windows 10 Pro.

## Results

### Radiomorphologic characteristics of NSC

The most common type of cranial dysmorphology was sphenocephaly (51/131 cases). Other types were: clinocephaly (38 cases), bathrocephaly (25 cases), dolichocephaly (12 cases) and leptocephaly (5 cases). Of all types of cranial deformation, sphenocephaly, clinocephaly and bathrocephaly accounted together for 87% of the studied population. Sphenocephaly patients were the youngest group, their mean age was 4.13 ± 1.95 months.

In terms of the pattern of sagittal suture fusion, the middle portion was fused the most often (125/131 cases). An association of age with the pattern of fusion was observed: the average age of children presenting the isolated M pattern was 3.74 months, the AM pattern 4.37 months, the MP pattern 5.74 months, and the AMP pattern — 7.24 months.

As for morphologic features, frontal bossing was the most common (120/131 cases). Other typical features like sagittal ridge (88/131) and occipital bullet (89/131) occurred with comparable frequency, but less often than bossing.

Finally, from the CSF alterations, the widening of the interhemispheric fissure was the most frequently observed element (116/131 cases). Frontal subarachnoid dilatation (81/131) or ventriculomegaly (88/131) were comparatively common. An isolated ventriculomegaly was found in 5/131 cases. In 9/131 cases no CSF alterations were observed. Detailed data are presented in Table 1 and the correlations between particular characteristics in Table 2.

**Table 1:** Radiomorphologic characteristics of NSC according to shape of the skull. Abbreviations in the text

			spheno	clino	bathro	dolicho	lepto	Total
Sagittal suture fusion	Portion involved	Middle portion (M)	50	37	24	11	3	125
		Posterior portion (P)	23	33	10	9	3	78
		Anterior portion (A)	17	14	13	7	4	55
	Pattern of fusion	MP	15	21	2	4	0	42
		AMP	7	11	8	5	2	33
		M	18	3	10	1	0	32
		AM	10	2	4	1	1	18
		P	1	0	0	0	1	2
		A	0	0	1	1	1	3
		AP	0	1	0	0	0	1
Morphologic features	General	Frontal bossing (B)	47	36	21	11	5	120
		Occipital bullet (O)	38	25	16	8	2	89
		Sagittal ridge (S)	39	26	13	7	3	88
	Combination of features	SOB	26	19	4	6	1	56
		SB	11	6	7	1	2	27
		OB	9	5	9	2	1	26
		B	1	6	1	2	1	11
		SO	1	1	2	0	0	4
		O	2	0	1	0	0	3
		S	1	0	0	0	0	1
		nil	0	1	1	1	0	3
CSF spaces alterations	General	Interhemispheric fissure (I)	47	35	23	9	2	116
		Ventriculomegaly (V)	32	28	18	7	3	88
		Frontal subarachnoid space (F)	32	26	16	7	0	81
	Combination of alterations	FIV	21	18	12	6	0	57
		IV	10	9	5	1	1	26
		FI	11	7	4	1	0	23
		I	5	1	2	1	1	10
		V	1	1	1	0	2	5
		F	0	1	0	0	0	1
		FV	0	0	0	0	0	0
		nil	3	1	1	3	1	9

**Table 2:** Correlations between radiomorphologic characteristics, sex and age. Correlation coefficients presented in the table are significant with *P* < 0.05. *NS*, not significant

	Shape of skull	Pattern of fusion	Morphologic features	CSF alterations
Age	*r* = 0.32 *P* = 0.0002	*r* = −0.30 *P* = 0.0004	NS	NS
Sex	NS	NS	NS	NS
Shape of skull	–	*V* = 0.37 *P* < 0.0001	NS	*V* = 0.27 *P* = 0.0412
Pattern of fusion	*V* = 0.37 *P* < 0.0001	–	NS	NS
Morphologic features	NS	NS	–	NS
CSF alterations	*V* = 0.27 *P* = 0.0412	NS	NS	–

### Cluster analysis

**Fig. 3:**
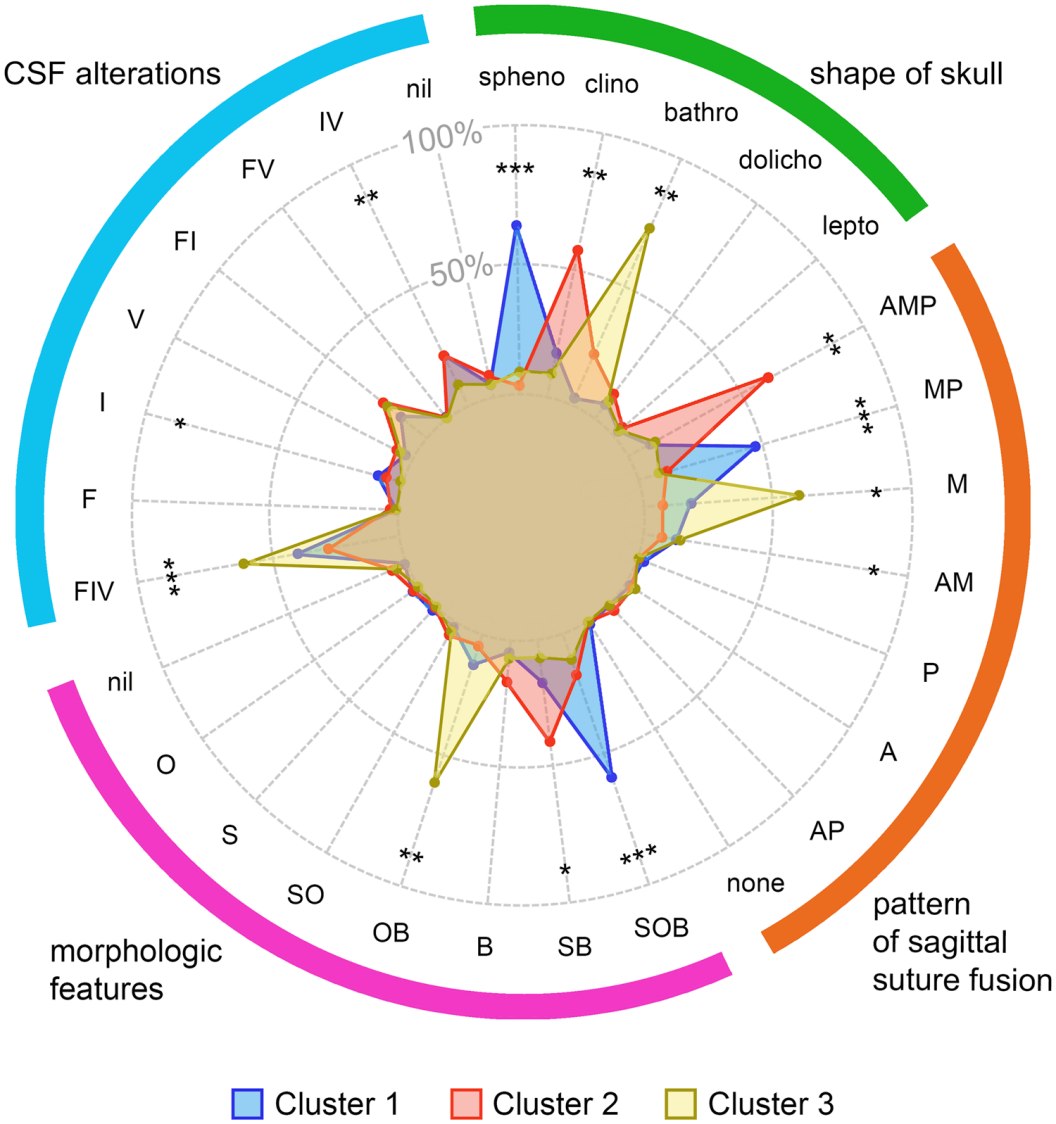
Radiomorphologic profiles of NSC represented by 3 different clusters. Radar plot shows the combinations of radiomorphologic characteristics per cluster for each assessed category. Spoke lengths represent the percentages of the studied characteristics per cluster. Significance levels are presented with asterisks. *P < 0.05; **P < 0.01; and ***P < 0.001

K-modes clustering revealed 3 distinct clusters with the highest internal similarity. A detailed distribution of assessed radiomorphologic characteristics is shown in Fig. 3 and Supplementary Table 1.

Cluster 1 was the largest cluster and represented 57.2% of NSC patients. Mean age was 5.06 months, SD 2.46. Patients in this cluster presented mostly sphenocephalic type of cranial deformity (64% of cases), but clinocephaly was also represented (22%). The most common pattern of sagittal suture fusion was MP (48%), followed by M and AM. In 63%, all the three morphologic features were present (SOB). CSF alterations were mainly represented by FIV, which was found in 47% of patients.

Cluster 2 comprised 24.4% of patients (mean age 6.47 months, SD 2.56). This cluster was defined by the most common occurrence of clinocephaly (59%). A prominent sagittal ridge combined with a frontal bossing (SB) were dominant morphologic features and amounted to 43%. The AMP pattern of sagittal suture fusion was the most common (62%) and FIV was a dominant combination of CSF alterations (31%).

Cluster 3 was the smallest cluster, representing 18.3% of the patients. Mean age was 5.15 months, SD 2.29. Unlike the remaining two clusters, in terms of the skull shape, this cluster was strongly dominated by the bathrocephalic type of cranial deformation (71%). The most common pattern of sagittal suture closure was the fusion in its middle portion (M, 58%). The assessment of morphologic features and CSF alterations showed OB and FIV as dominant combinations (both 62%).

A comparative analysis of clusters revealed no significant differences between boys and girls (*P* = 0.5171). The age difference was significant only between clusters 1–2 (overall *P* = 0.0265).

The clusters revealed significant correlations with radiomorphologic characteristics – the strongest with the skull shape (V = 0.58, *P* < 0.0001) and morphologic features (V = 0.50, *P* < 0.0001). A statistically significant, but weaker correlation was observed between clusters and the pattern of sagittal suture fusion (V = 0.47, *P* < 0.0001). CSF alterations did not significantly correlate with the clusters (*P* = 0.3585).

## Discussion

Morphologic variability of NSC has been reflected in numerous classifications based on different morphologic and radiologic criteria, such as skull shape [12], pattern of sagittal suture fusion [16], type of cranial vault deformity [22], the presence of leading morphologic features (frontal bossing, coronal constriction, temporal or occipital protrusions) or recently, the three-dimensional measurements [23]. Basing on the constant clinical appearance, Schmelzer et al. in 2007 proposed four patterns of calvarial dysmorphology with one dominant feature [24]. However, the patterns did not explain all morphologic dissimilarities and in the following years some new classifications were developed by David et al. [17] or Sakamoto et al. [22].

In 2022, Diab et al. attempted a novel classification and approach to diagnosis of NSC [18], incorporating the alterations of cerebrospinal fluid (CSF) spaces, discussed since the late 1980s of the twentieth century by Maytal et al. [25], Collman et al. [26, 27], Odita [28] and Chadduck et al. [29]. The prevalence of various morphologic markers (clinical appearance, radiologic neurologic features and region of sagittal suture fusion) described in the paper by Diab et al. is the latest attempt to combine distinct morphologic categories into one coherent whole. In that interesting paper, the authors presented a relation between morphologic markers and the age of NSC patients, thus we could learn a lot about each marker separately. In our opinion, however, the paper lacks an identification of groups of patients similar in terms of morphology, which could possibly allow a reliable definition of the morphology of NSC as a desired biomarker of NSC.

Cluster analysis we performed may facilitate this approach. It enabled a distinction of the 3 most common combinations of NSC characteristics that we called “radiomorphologic profiles” (Table 3). In each cluster, a dominance of a different feature or combination of features was observed, which supports the separateness of the profiles represented by the clusters.

**Table 3:** Radiomorphologic profiles of NSC including the mean age and the most common combination of the studied NSC characteristics in each category. *P*, p-value; *SD*, standard deviation

	Profile 1	Profile 2	Profile 3	*P*
Mean age ± SD	5.06 ± 2.46	6.47 ± 2.56	5.15 ± 2.29	0.0265
Shape of skull	sphenocephaly	clinocephaly	bathrocephaly	< 0.0001
Pattern of sagittal suture fusion	MP	AMP	M	< 0.0001
Morphologic features	SOB	SB	OB	< 0.0001
CSF alterations	FIV	FIV	FIV	0.3585

**Fig. 4:**
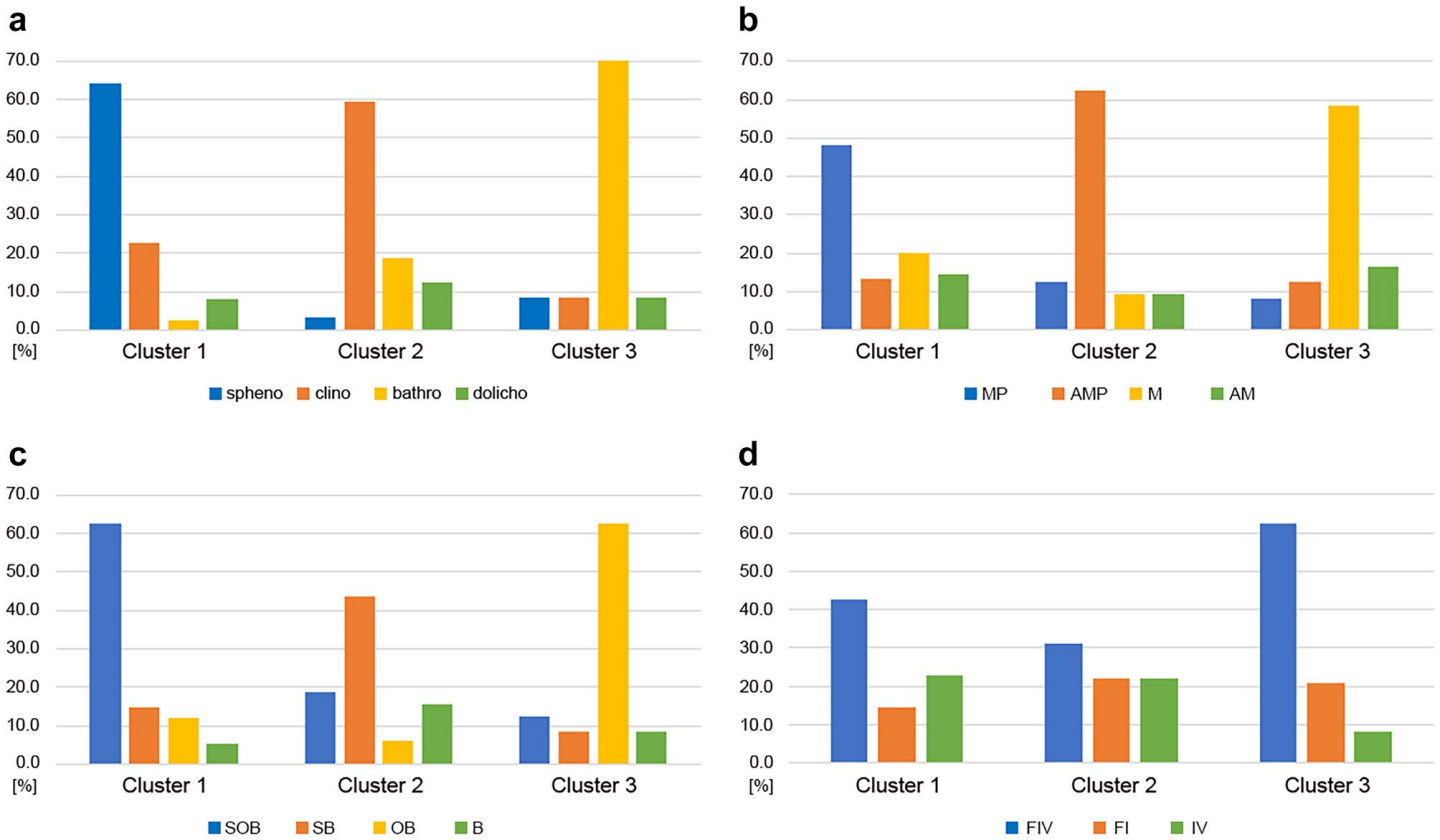
Distribution of features/combinations of features in each cluster: shape of skull **a**; pattern of sagittal suture fusion **b**; morphologic features **c**; CSF alterations **d**

Cluster 1 was dominated by sphenocephalic patients. In the remaining clusters, sphenocephaly did not exceed the level of 10%, while other skull shapes were observed more frequently. The dominance of clinocephaly in cluster 2 was not as high as that of sphenocephaly in cluster 1, but none of the remaining shapes reached the level of 20% in cluster 2. The greatest predominance of one type was observed in cluster 3, where bathrocephaly was present in 71%, and the remaining types did not exceed the level of 10% (Fig. 4a). It should be noted, however, that cluster 3 was the smallest cluster.

In terms of sagittal suture fusion pattern, the middle portion was fused the most often (94–96%) in each cluster; however, the involvement of the remaining parts was different between clusters. In cluster 1, the posterior portion was fused twice as often as the anterior portion, in cluster 2 both other portions — anterior and posterior, were fused in a comparably high percentage. However, in cluster 3 the involvement of both other portions was also comparable, but much lower than in cluster 2 (Supplementary Fig. 1a). This distribution probably results in a clear dominance of one of the patterns in each cluster. The dominant pattern in cluster 1 was MP — the most common across the studied population (Fig. 4b). In cluster 2, the AMP pattern clearly dominated, and the remaining patterns were represented at the level of 9–12% each. In cluster 3, a definite dominance of a single pattern (M) was observed, and the remaining patterns reached the level of 8–17%. These observations indicate that patients showing high morphologic similarity do not always present the same pattern of sagittal suture fusion, which was depicted in the study by Villavisanis et al. [8]. On the other hand, considering that the average age of children with an isolated pattern M was the lowest, a hypothesis about possible directions of sagittal fusion presented by Heuzé et al. seems authenticated. In the paper from 2010 the authors stated that the potential direction of evolution of the M pattern is the AMP pattern, and the transition stages are the MP or AM patterns [16]. Other authors reported that posterior portion of sagittal suture fuses more frequently in older age [18]. These findings seem confirmed by the average ages calculated in our patients.

The analysis of the occurrence of morphologic features also showed significant differences between the clusters. Frontal bossing was the most frequently observed feature across the clusters (84–95% of cases). Cluster 1 showed a comparably high occurrence of two other features: sagittal ridge and occipital bullet. Significantly lower incidence of occipital bullet was observed in cluster 2, and cluster 3 was characterized by a small percentage of sagittal ridge (Supplementary Fig. 1b). SOB was the most common combination and dominated in cluster 1, but did not exceed 20% in the remaining clusters (Fig. 4c). It is worth noting that the most common combination in cluster 3 was OB, which results from the fact that the occurrence of sagittal ridge in cluster 3 was as low as 25%. In turn, the dominant pattern in cluster 2 was SB, which is related to the prevalence of occipital bullet in clinocephaly at the level of 34%. Similarly, to suture fusion patterns, the occurrence of morphologic features importantly influenced the allocation to clusters, but as a marker stand-alone it did not correlate with other NSC characteristics assessed in the study.

In terms of CSF alterations, the most common feature in each cluster was the widening of interhemispheric fissure (81–92%). Ventriculomegaly was second most common feature in cluster 1 (68%) and cluster 2 (59%) and widening of frontal subarachnoid space was second most common feature in cluster 3 (83%) (Supplementary Fig. 1c). Given this distribution, it seems natural that FIV was the most common combination in all clusters, but in cluster 3 it was clearly dominant before FI and IV (Fig. 4d). Cluster 2 was the most balanced — the occurrence of all 3 most common patterns was comparable.

### Clinical significance

A reliable clinical assessment of NSC requires comparable groups of patients. A direct comparison between single morphologic criteria yields few significant correlations (see Table 2). It seems that the type of deformation (shape) of the skull is the most important, because it was the only one that significantly correlated with age, pattern of sagittal suture fusion and CSF alterations, and it also showed the highest correlation with radiomorphologic profiles.

Distinguishing the profiles has in our opinion the advantage of confirming the radiomorphologic dissimilarity between apparently similar subjects. We believe that the designation of groups of patients radiomorphologically similar is in the interest of future evaluation of treatment effects of NSC. Today, the outcomes are assessed for the whole group of NSC based on various criteria: morphologic [30], neurodevelopmental [31, 32] or cosmetic [33, 34]. Current analyses of treatment results take into account such variables as patients’ age or the surgical technique used (cranial vault remodeling, endoscopic or spring assisted surgery) [35–38]. All these analyses, however, treat the NSC as a whole, without taking into account a morphologic variability within this entity. Our study confirms in a statistically significant way that the separation of homogeneous groups of patients has a scientific basis, and the conducted analysis proved that among the examined characteristics, it is the shape of skull that is the most reliable differentiating factor.

### Study limitations

This study presents some limitations. The size of the studied NSC population is, to our knowledge, appropriate, regarding other analyses [8, 32]; however, the size of particular subgroups may be suboptimal for reliable statistical analysis. Another limitation is cluster analysis. In unsupervised analysis, the optimal number of clusters should be established in advance. In our population, this number was dictated by a multivariate way of data analysis — in each of the studied characteristics, 3 or 4 combinations clearly dominated, which turned out to be typical for similar groups of patients. Finally, the assessment of analyzed radiomorphologic characteristics may also be limited due to the increasing tendency to limit the role of computed tomography in the diagnostics of NSC.

## Conclusions

The NSC is a mosaic of radiologic and morphologic features. The internal diversity of NSC results in dissimilar groups of patients defined by unique combinations of radiomorphologic characteristics, from which the skull shape is the most differentiating factor. Radiomorphologic profiles we distinguished in this study support the idea of clinical trials targeted at more selective outcomes assessment.

### Supplementary Information

Below is the link to the electronic supplementary material.

Supplementary file1 (PDF 180 KB)

## Data Availability

The data belong to Bogdanowicz Memorial Hospital for Children in Warsaw and are not available to share unless in the form included in the manuscript and supplementary materials.

## References

[1] Morriss-Kay GM, Wilkie AO (2005). Growth of the normal skull vault and its alteration in craniosynostosis: insights from human genetics and experimental studies. J Anat.

[2] Wu R, Nie J, Abraham P (2021). Neurologic characterization of craniosynostosis: can direct brain recordings predict language development?. J Craniofac Surg.

[3] Chieffo DPR, Arcangeli V, Bianchi F (2020). Single-suture craniosynostosis: is there a correlation between preoperative ophthalmological, neuroradiological, and neurocognitive findings?. Childs Nerv Syst.

[4] FDA-NIH Biomarker Working Group (2016) BEST (Biomarkers, EndpointS, and other Tools) Resource. Silver Spring (MD): Food and Drug Administration (US); Bethesda (MD): National Institutes of Health (US). Available at: https://www.ncbi.nlm.nih.gov/books/NBK326791 27010052

[5] Boltshauser E, Ludwig S, Dietrich F, Landolt MA (2003). Sagittal craniosynostosis: cognitive development, behaviour, and quality of life in unoperated children. Neuropediatrics.

[6] Wu RT, Abraham P, Nie J (2018). Abstract: direct brain recordings in craniosynostosis can predict future language development. Plast Reconstr Surg Glob Open.

[7] Aldridge K, Collett BR, Wallace ER (2017). Structural brain differences in school-age children with and without single-suture craniosynostosis. J Neurosurg Pediatr.

[8] Villavisanis DF, Blum JD, Cho DY (2022). Degree of sagittal suture fusion, cephalic index, and head shape in nonsyndromic sagittal craniosynostosis. J Craniofac Surg.

[9] Galiay L, Hennocq Q, Cross C (2022). Management of sagittal craniosynostosis: morphological comparison of eight surgical techniques. Br J Oral Maxillofac Surg.

[10] Bradford PS, Ishaque M, Shaffrey E (2021). Evolution of surgical management of sagittal synostosis. J Craniofac Surg.

[11] Shipster C, Hearst D, Somerville A, Stackhouse J, Hayward R, Wade A (2003). Speech, language, and cognitive development in children with isolated sagittal synostosis. Dev Med Child Neurol.

[12] Di Rocco F, Gleizal A, Szathmari A, Beuriat PA, Paulus C, Mottolese C (2019). Sagittal suture craniosynostosis or craniosynostoses. The heterogeneity of the most common premature fusion of the cranial sutures. Neurochirurgie.

[13] Captier G, Bigorre M, Rakotoarimanana JL, Leboucq N, Montoya P (2005) Etude des variations morphologiques des scaphocéphalies. Implication pour leur systématisation [Study of the morphologic variations of the scaphocephaly. Deduction for their systematisation]. Ann Chir Plast Esthet 50(6):715–722. 10.1016/j.anplas.2005.06.001 10.1016/j.anplas.2005.06.001 16084003

[14] Pfaff MJ, Fenton R, Mittal A (2023). The clinical significance of clinocephaly in late-presentation sagittal craniosynostosis. Cleft Palate Craniofac J.

[15] Golinko M, Bonfield C (2022). Clinical staging of craniosynostosis: a call for investigation and collaboration. Childs Nerv Sys.

[16] Heuzé Y, Boyadjiev SA, Marsh JL (2010). New insights into the relationship between suture closure and craniofacial dysmorphology in sagittal nonsyndromic craniosynostosis. J Anat.

[17] David L, Glazier S, Pyle J, Thompson J (2009). Argenta L (2009) Classification system for sagittal craniosynostosis. J Craniofacial Surg.

[18] Diab J, Flapper W, Grave B, Abou-Hamden A, Anderson P, Moore M (2022). The many faces of sagittal synostosis: a novel classification and approach to diagnosis. J Craniofacial Surg.

[19] Fairman D, Horrax G (1949). Classification of craniostenosis. J Neurosurg.

[20] Cohen MM, Cohen MM, MacLean RE (2000). History, terminology, and classification of craniosynostosis. Craniosynostosis: diagnosis, evaluation, and management.

[21] Huang Z, Lu H, Motoda H, Luu H (1997). A fast clustering algorithm to cluster very large categorical data sets in data mining. KDD: techniques and applications.

[22] Sakamoto Y, Nakajima H, Tamada I, Miwa T, Kishi K, Yoshida K (2014). New pathogenesis and the classification in scaphocephaly. J Plast Surg Hand Surg.

[23] Blum JD, Cho DY, Cheung L (2022). Making the diagnosis in sagittal craniosynostosis-it’s height, not length, that matters. Childs Nerv Syst.

[24] Schmelzer RE, Perlyn CA, Kane AA, Pilgram TK, Govier D, Marsh JL (2007). Identifying reproducible patterns of calvarial dysmorphology in nonsyndromic sagittal craniosynostosis may affect operative intervention and outcomes assessment. Plast Reconstr Surg.

[25] Maytal J, Alvarez LA, Elkin CM, Shinnar S (1987). External hydrocephalus: radiologic spectrum and differentiation from cerebral atrophy. AJR Am J Roentgenol.

[26] Collmann H, Sörensen N, Krauss J, Mühling J (1988). Hydrocephalus in craniosynostosis. Childs Nerv Syst.

[27] Collmann H, Sörensen N, Krauss J (2005). Hydrocephalus in craniosynostosis: a review. Childs Nerv Syst.

[28] Odita JC (1992) The widened frontal subarachnoid space. A CT comparative study between macrocephalic, microcephalic, and normocephalic infants and children. Childs Nerv Syst 8(1):36–39. 10.1007/BF00316560 10.1007/BF00316560 1576605

[29] Chadduck WM, Chadduck JB, Boop FA (1992). The subarachnoid spaces in craniosynostosis. Neurosurgery.

[30] Malde O, Cross C, Lim CL (2020). Predicting calvarial morphology in sagittal craniosynostosis. Sci Rep.

[31] Kalmar CL, Lang SS, Heuer GG (2022). Neurocognitive outcomes of children with non-syndromic single-suture craniosynostosis. Childs Nerv Syst.

[32] Junn AH, Long AS, Hauc SC et al (2023) Long-term neurocognitive outcomes in 204 single-suture craniosynostosis patients [ahead of print]. Childs Nerv Syst. 10.1007/s00381-023-05908-1 10.1007/s00381-023-05908-1 36877207

[33] Whitaker LA, Pashayan H, Reichman J (1981). A proposed new classification of craniofacial anomalies. Cleft Palate J.

[34] Millesi M, Preischer M, Reinprecht A (2021). Do standard surgical techniques lead to satisfying aesthetic results in nonsyndromic sagittal suture synostosis?. J Neurosurg Pediatr.

[35] Dempsey RF, Monson LA, Maricevich RS (2019). Nonsyndromic craniosynostosis. Clin Plast Surg.

[36] Marupudi NI, Reisen B, Rozzelle A, Sood S (2022). Endoscopy in craniosynostosis surgery: evolution and current trends. J Pediatr Neurosci.

[37] Ou Yang O, Marucci DD, Gates RJ (2017). Analysis of the cephalometric changes in the first 3 months after spring-assisted cranioplasty for scaphocephaly. J Plast Reconstr Aesthet Surg.

[38] Alperovich M, Runyan CM, Gabrick KS (2021). Long-term neurocognitive outcomes of spring-assisted surgery versus cranial vault remodeling for sagittal synostosis. Plast Reconstr Surg.

