# Supplementary material for: Radiomorphologic profiles of nonsyndromic sagittal craniosynostosis
Source: Childs Nerv Syst. 2023 May 27;39(11):3225–33. doi: 10.1007/s00381-023-05998-x (PMC10643241; doi:10.1007/s00381-023-05998-x)
Supplement: Supplementary file 1 — Supplementary file1 (PDF 180 KB) [file 381_2023_5998_MOESM1_ESM.pdf]

Title: Radiomorphologic profiles of nonsyndromic sagittal craniosynostosis

Authors:
Tymon Skadorwa, ORCID: 0000-0002-3775-3968
Olga Wierzbieniec, ORCID: 0000-0002-1926-6752
Kamila Sośnicka, ORCID: 0009-0002-6764-8306
Klaudia Podkowa, ORCID: 0009-0008-1391-6065

Supplementary Table 1

		Cluster 1	Cluster 2	Cluster 3
	n	75	32	24
	age±SD (months)	5.06±2.46	5.06±2.46	5.06±2.46
Shape of skull	sphenocephaly	48	1	2
	clinocephaly	17	19	2
	bathrocephaly	2	6	17
	dolichocephaly	6	4	2
	leptocephaly	2	2	1
Pattern of sagittal suture fusion	AMP	10	20	3
	MP	36	4	2
	M	15	3	14
	AM	11	3	4
	P	2	0	0
	A	1	1	1
	AP	0	1	0
	none	1	0	0
Clinical features	SOB	47	6	3
	SB	11	14	2
	B	4	5	2
	OB	9	2	15
	SO	1	2	1
	S	1	0	0
	O	2	1	0
	nil	0	2	1
CSF alterations	FIV	32	10	15
	F	0	1	0
	I	8	2	0
	V	2	2	1
	FI	11	7	5
	FV	0	0	0
	IV	17	7	2
	nil	5	3	1

Suppl. Tab. 1 Distribution of radiomorphologic characteristics according to clusters.

Supplementary Figure 1

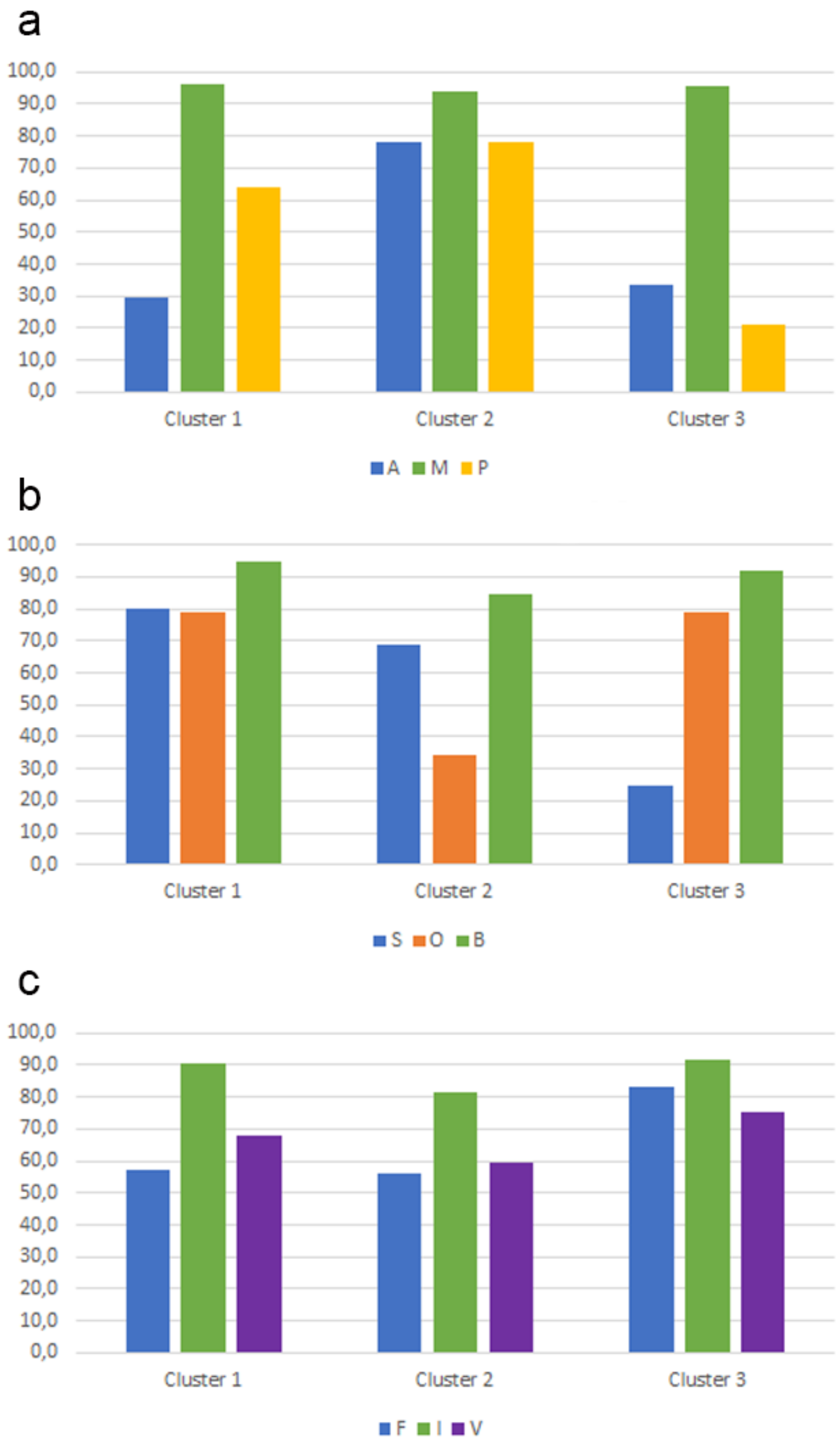

Suppl. Fig. 1 Percentage distribution of radiomorphologic characteristics according to clusters.
